# Regulation of Paneth Cell Function by RNA-Binding Proteins and Noncoding RNAs

**DOI:** 10.3390/cells10082107

**Published:** 2021-08-17

**Authors:** Hee K. Chung, Lan Xiao, Krishna C. Jaladanki, Jian-Ying Wang

**Affiliations:** 1 Cell Biology Group, Department of Surgery, University of Maryland School of Medicine, Baltimore, MD 21201, USA; HKChung@som.umaryland.edu (H.K.C.); LXiao@som.umaryland.edu (L.X.); kcjaladanki@gmail.com (K.C.J.); 2 Baltimore Veterans Affairs Medical Center, Baltimore, MD 21201, USA; 3 Department of Pathology, University of Maryland School of Medicine, Baltimore, MD 21201, USA

**Keywords:** Paneth cells, RNA-binding proteins, noncoding RNAs, microRNAs, circular RNAs, long noncoding RNAs, epithelial homeostasis

## Abstract

Paneth cells are specialized intestinal epithelial cells that are located at the base of small intestinal crypts and play a vital role in preserving the gut epithelium homeostasis. Paneth cells act as a safeguard from bacterial translocation across the epithelium and constitute the niche for intestinal stem cells in the small intestine by providing multiple niche signals. Recently, Paneth cells have become the focal point of investigations defining the mechanisms underlying the epithelium-microbiome interactions and pathogenesis of chronic gut mucosal inflammation and bacterial infection. Function of Paneth cells is tightly regulated by numerous factors at different levels, while Paneth cell defects have been widely documented in various gut mucosal diseases in humans. The post-transcription events, specific change in mRNA stability and translation by RNA-binding proteins (RBPs) and noncoding RNAs (ncRNAs) are implicated in many aspects of gut mucosal physiology by modulating Paneth cell function. Deregulation of RBPs and ncRNAs and subsequent Paneth cell defects are identified as crucial elements of gut mucosal pathologies. Here, we overview the posttranscriptional regulation of Paneth cells by RBPs and ncRNAs, with a particular focus on the increasing evidence of RBP HuR and long ncRNA H19 in this process. We also discuss the involvement of Paneth cell dysfunction in altered susceptibility of the intestinal epithelium to chronic inflammation and bacterial infection following disrupted expression of HuR and H19.

## 1. Introduction

The epithelium of mammalian intestine self-renews rapidly and its surfaces are exposed to a wide variety of luminal noxious substances and are colonized by complex microbiota. Most bacteria perform beneficial functions, but they can also alter the intestinal epithelium homeostasis and threaten host health upon tissue invasion [1,2]. The protective intestinal mucosal barrier against luminal toxins, antigens, and bacterial invasion is a complex process consisting of multiple elements, including mucus layer, epithelial layer, and immune defense systems [1,3]. Residing at the bottom of the small intestinal crypts, Paneth cells are highly specialized secretory cells that are critical for maintaining integrity of the small intestinal epithelium by sustaining host defense against enteric pathogens [4,5] and preserving the health of intestinal stem cell niches [6,7]. Paneth cells synthesize an ample number of antibacterial proteins or peptides, including lysozymes, α-defensins, C-type lectins, phospholipase A2, RegIII, MMP-7, CRIP, and xanthine oxidase [5,8,9]. These secretory substances within Paneth cells are initially assembled together and packaged into dense core granules by an endoplasmic reticulum (ER) and Golgi complex network and can be rapidly released from the apical side of the cell into the lumen to protect the epithelium from bacteria, fungi, protozoans, and virus infections [5,6,10]. Paneth cells also provide multiple secreted (Wnts, EGF) and surfaced-bound (Notch ligand) niche signals essential for intestinal stem cell (ISC) maintenance and function in response to pathophysiologic stress [11,12]. Nonetheless, defects in Paneth cells occur commonly in different gut mucosal diseases and compromise the epithelial protection and constant renewal [11,13,14].

Posttranscriptional controls, especially altered mRNA stability and translation by RNA-binding proteins (RBPs) and noncoding RNAs (ncRNAs), are major mechanistic events by which mammalian cells regulate gene expression [15]. After mRNAs are transcribed from DNAs, they are subject to several processing and regulatory steps. Among these processes, alterations in mRNA turnover and translation are mainly governed by the association of specific mRNA sequences (*cis*-elements) with two important types of trans-acting factors such as RBPs and ncRNAs [16,17]. RBPs directly interact with target mRNAs via AU-rich elements (AREs), or GU-rich elements (GREs) distributed in the 3′-untranslated regions (UTRs) and/or coding regions (CRs), and these associations alter mRNA turnover and translational efficiency positively or negatively [18]. On the other hand, ncRNAs, including microRNAs (miRNAs), long ncRNAs (lncRNAs), and circular RNAs (circRNAs) are intimately involved in every level of gene regulation such as transcriptional and posttranscriptional processes, chromatin remodeling, and protein metabolism [16,19,20]. Increasing evidence indicates that RBPs and ncRNAs are a novel class of master posttranscriptional modulators of gut epithelial homeostasis and that disrupted regulation of RBPs such as HuR and ncRNAs including lncRNA *H19* results in Paneth cell defects. Dysfunction of Paneth cells is involved in the pathogenesis of various gut epithelial disorders including mucosal infection and inflammatory bowel diseases (IBD) [16,21]. Since there are already several excellent reviews that summarize recent advance of Paneth cell biology in general [22,23,24], this review is highly focused on the major roles RBPs and ncRNAs play in the regulation of Paneth cell function and further discuss in some detail the mechanisms by which RBPs and ncRNAs influence the expression of target genes at the posttranscription level.

## 2. Paneth Cells in the Intestinal Epithelium Homeostasis

Paneth cells were first identified in 1888 by Josef Paneth as columnar gut epithelial cells possessing prominent eosinophilic granules in their cytoplasm [13]. Development of Paneth cells is evident in both small intestine and colon at an early gestational age, but they move down to the crypts of the small intestine and disappear in colonic mucosa after birth. Unlike most intestinal epithelial cells (IECs) that are quickly turned over within a few days, Paneth cells can persist for months in healthy individuals. Paneth cells are interspersed between ISCs and constitute Paneth cell/ISC niche essential for continuous mucosal growth [14]. In some pathological conditions such as chronic inflammation, however, Paneth cells abnormally arise in other organs including the esophagus and colon (termed as metaplastic Paneth cells), although the role of metaplastic Paneth cells remains to be elucidated [24]. As summarized in Table 1, emerging evidence shows that Paneth cells are crucial for sustaining homeostasis of the intestinal epithelium by enhancing the host defense and promoting constant epithelial renewal. Paneth cell dysfunction exhibits significant detrimental consequences, including diminished clearance of bacterial pathogens, impairment of ISC function, and development of mucosal inflammation [23,24,25].

### 2.1. Paneth Cells Enhance Epithelial Defense by Secreting Antimicrobial Peptides/Proteins

As specialized and secretory IECs in the small intestinal mucosa, Paneth cells produce a variety of antimicrobial peptides and proteins [25,30]. Because the surfaces of the small intestinal mucosa are constantly awash with bacteria and their products, Paneth cells continuously synthesize and release antimicrobials at a baseline rate via granule secretion mechanism, with increased amounts secreted on maximum stimulation. In response to bacterial infection, for example, these antimicrobial-rich granules in Paneth cells are released into the crypt lumen, where increased accumulations of antimicrobials prevent bacterial invasion of the crypt and protect the epithelium against infections by enteric pathogens [37,40]. Many antimicrobial proteins/peptides destroy their target bacteria by impairing integrity of the membrane, but some peptides/proteins specifically repress bacterial cell wall synthesis through the association with lipid II [41]. Defensins are a major family of antimicrobial peptides in mammals, and Paneth cells produce many antimicrobial peptides and proteins that are evolutionally related to α-defensins. The α-defensins are only expressed in Paneth cells in mice and contributes to the enteric innate immunity [25,26]. Availability of enteric α-defensins and its impact on epithelial defense are markedly linked to Paneth cell function and homeostasis. Mice overexpressing the *Defa5/6* gene exhibit Paneth cell dysfunction, along with a significant alteration in the composition of resident ileal microbiota, similar to those observed in IBD patients [28]. As altered microflora in human is implicated in pathogenesis of IBD, the quantity and the repertoire of Paneth cell α-defensins and other-related genetic products in such condition alter gut mucosal defense and affect the epithelial integrity by constituting a healthy microbiota or by increasing risk to inflammation and infections in genetically predisposed individuals [28,29,37,40].

Several studies have demonstrated the necessity of autophagy in Paneth cells, including formation of secretory granules, secretion of antimicrobial peptides and/or proteins, and control of host immunity [10,11,13,26,27]. As a conserved intracellular pathway, autophagy sequesters the cytoplasmic structures and pathogens involved in the process of degradation [31,42]. Autophagy is required for maintaining normal structure and function of various cellular organelles such as ER and mitochondria in Paneth cells, while lysozyme secretion by Paneth cells is mediated via secretory autophagy to limit intestinal bacterial infection [26,31]. Mutation of the autophagy-related genes (*Atgs*) leads to dysfunctional mitochondria and ER and subsequent defects in Paneth cells, thus enhancing the release of inflammatory cytokines in dextran sulfate sodium-induced colitis in mice and other pathological conditions [31,32,37,40,43]. Mice with hypomorphic IBD-associated allele *Atg16L1* exhibit reduced Paneth cells, which is associated with an inhibition of secretory granule formation, a decrease in lysozyme synthesis, and an increase in inflammatory cytokines by macrophages [32,33]. The *Atg16L1* deletion in mice causes abnormal alterations such as Paneth cell number, organoid assembly, granule structure, and secretion of antimicrobial peptides [4,29,32]. Loss of *Atg4B*, *Atg5*, or *Atg7* genes also results in Paneth cell defects; all these findings suggest that autophagy plays a critical role in the control of Paneth cell function [22,32].

### 2.2. Paneth Cells Regulate Intestinal Mucosal Growth by Interacting with Intestinal Stem Cells

The human small intestinal epithelium undergoes ~10^11^ mitoses/day and this rapid and dynamic turnover rate is driven by ISCs, which are tightly regulated by multiple factors at different levels [16,33]. ISCs divide daily and produce bipotent progenitors amplifying and differentiating into absorptive or secretory lineages [12,33]. Paneth cells create the niche for ISCs in the crypts and provide many secreted as well as surfaced-bound niche signals [11,12]. The Paneth cell niche is the microenvironment in which ISCs both reside and receive stimulations that determine their fate in vivo. Emerging evidence indicates that interactions between Paneth cells and ISCs in the intestinal crypts are essential for continuous and rapid intestinal epithelial renewal under various pathophysiological conditions [27,44]. Coculturing of sorted Paneth cells with ISCs dramatically enhances intestinal organoid formation and growth [12], whereas Paneth cell defects results in ISC dysfunction and leads to an inhibition of intestinal mucosal growth [12,27]. In the case of Paneth cell ablation, enteroendocrine and Tuft cells and intestinal stromal cells can also support ISC function [11,45,46].

## 3. Regulation of Paneth Cell Function by RBPs

RBPs are a big family of proteins that contain functional and structural motifs including dsRNA binding domain and RNA recognition motif (RRM) and play a pivotal role in processing of newly transcribed mRNAs [16,43]. RBPs directly associate with specific subsets of mRNAs and regulate mRNA splicing, nuclear degradation/exportation, stability, and translation. Many RBPs, such as tristetraprolin (TTP), AU-binding factor 1 (AUF1), CUG-binding protein 1 (CUGBP1), BRF1, and KH-domain RNA binding protein (KSRP), directly interact with target mRNAs via AREs or GREs and promote the decay of transcripts via the mRNA recruitment to the processing bodies (P-bodies) and/or proteasome where mRNAs are sorted for degradation [16,47]. On the other hand, the Hu/embryonic lethal and abnormal vision (ELAV) group of RBPs consists of three specific neuronal members, including HuB (ELAVL2), HuC (ELAVL3) and HuD (ELAVL4), and one ubiquitous member HuR (ELAVL1), and they increase the translation and stability of target mRNAs in general [48]. Notable changes in the binding affinity of RBPs for their target transcripts, mutations and defects in their binding sites, and disruption of RBP expression and subcellular localization have been described in different diseases such as IBD and colonic cancers in humans [35,39,47]. Table 2 summarizes recent studies showing that RBPs function as key regulators of Paneth cell function in response to various stressful environments [27,32,43].

### 3.1. HuR Is Essential for Paneth Cell Function

HuR is a well-studied RBP and contains two N-terminal RRMs by which HuR directly binds with high specificity and affinity to AREs located in 3′-UTR of numerous labile mRNAs [16,34]. In most cases, HuR interaction with mRNAs induces the stability and translation of target transcripts, thereby sustaining the epithelium homeostasis [16,32,34]. Intestinal epithelium tissue-specific HuR deletion (IE-HuR^−/−^) in mice causes defective Paneth cells in the small intestinal mucosa [27]. Immunostaining assays show that lysozyme-positive cells are typically distributed at the crypt base in control littermates, but the amount of these lysozyme-positive cells significantly reduces in IE-HuR^−/−^ mice when compared with littermate mice. Total number of lysozyme granules per Paneth cell also lowers remarkably in the HuR-deficient mucosal tissues. Conversely, targeted deletion of HuR in IECs fails to change the differentiation and function of Goblet cells, since there are no significant differences in the levels of Goblet cells in the mucosa between IE-HuR^−/−^ mice and control littermates as examined by both Alcian blue staining and mucin-2 immunostaining assays.

In an ex-vivo model, there also are evident defects in Paneth cells in primarily cultured intestinal organoids derived from IE-HuR^−/−^ mice [27]. Targeted deletion of HuR also remarkably suppresses growth of the organoids, as intestinal organoids generated from the IE-HuR^−/−^ mice are smaller and contain fewer buds when compared to those observed in the organoids from littermate mice. Furthermore, the rate of DNA synthesis in the organoids generated from HuR-deficient mice is also decreased, as indicated by the reduced population of BrdU-positive cells. Like the findings in vivo, Paneth cells in the intestinal organoids derived from littermate mice are greatly enriched, but they dramatically decrease and are almost invisible in the intestinal organoids isolated from IE-HuR^−/−^ mice. This defect in Paneth cells by HuR knockout is accompanied by inhibited autophagic clearance, since HuR silencing also prevents autophagy activation induced by treatment with a pharmacologic inducer rapamycin in cultured IECs. Interestingly, IBD patients show reduced levels of both HuR and lysozyme-positive cells in the small intestinal mucosa, compared with tissues from control individuals. The levels of HuR and Paneth cells in human ileal mucosal tissue samples from IBD patients are almost undetectable, which is along with massive amount of mucosal erosions/injury, inflammation, and barrier dysfunction [27,32]. Together, these findings strongly suggest that HuR plays a key role in maintaining Paneth cell function in the small intestinal mucosa.

### 3.2. HuR Regulates Paneth Cells by Altering TLR2 Membrane Distribution

Toll-like receptors (TLRs) are primarily localized at the apical surface of villi in the intestinal epithelium and are necessary for function of Paneth cells [49,60]. Targeted deletion of HuR results in defects in Paneth cells by inactivating TLR2 activity, since TLR2 acts as a key sensor in Paneth cells and IE-HuR^−/−^ mice do not have the apical localization of TLR2 in the intestinal mucosa as identified in littermate mice [27]. In mice with ablated HuR, TLR2 staining in the small intestinal mucosa disappears completely from apical surface, but it shows diffused distribution in the cytoplasm with great intensity in basal region of the intestinal epithelium. The most prominent differences between IE-HuR^−/−^ mice and littermates are the appearance of many regions of small punctate TLR2 foci in the HuR^−/−^ mice. These specific punctate areas are interspersed throughout entire cytoplasm of the HuR-deficient mucosal tissues and can be found in every IE-HuR^−/−^ mouse in general. Likewise, TLR2 localization in the colonic epithelium is also disrupted by HuR knockout, as demonstrated by a reduction in the apical straining of TLR2 in IE-HuR^−/−^ mice relative to control mice. Consistent with the in-vivo observations, TLR2 staining is typically observed at the luminal areas in primarily cultured organoids derived from littermate mice, but this specific TLR2 localization is suppressed by HuR deletion. TLR2 immunostaining at the luminal regions is dispersed in the intestinal organoids derived from IE-HuR^−/−^ mice. In contrast, HuR deletion does not affect subcellular localization of TLR4 in the intestinal epithelium.

HuR deletion in mice fails to modify the levels of whole cell TLR proteins in the mucosa of the small intestine. In wild type and littermate mice, TLR2 and TLR4 are predominantly expressed in cultured IECs and mouse gut mucosal tissues, although the levels of TLR6 and TLR1 proteins are very low or undetectable in the intestinal mucosa in vivo and in vitro [27]. There are no detectable differences in the abundances of TLR2 and TLR4 proteins in the intestinal mucosa between IE-HuR^−/−^ mice and littermates. Most notably, conditional HuR deletion in mice decreases the levels of ER chaperone protein canopy-3 (CNPY3) in the intestinal epithelium. The mucosal tissues obtained from IE-HuR^−/−^ mice also exhibits reduced levels of beclin-1 and IRGM but increased abundances of NLRX1, without affecting NOD1 content. In the studies conducted in cultured IECs, HuR silencing also reduces cellular CNPY3 protein, but it does not affect cellular levels of TLR4, TLR2, RIP2, PGRP-1α, MyD88, NOD1, NOD2, or gp96 proteins. These results indicate that reducing the HuR levels in the intestinal mucosa impairs the subcellular distribution of TLR2 and leads to Paneth cell dysfunction, but it fails to alter TLR2 whole-cell levels.

### 3.3. HuR Promotes TLR2 Subcellular Trafficking and Autophagy by Increasing CNPY3

CNPY3 protein is essential for the proper folding and subcellular distribution of TLR2 on the plasma membrane to carry out secretory autophagy in Paneth cells [26,27,49]. CNPY3 directly interacts with TLR2 and forms the CNPY3/TLR2 complexes, thus increasing proper folding and subcellular transportation and promoting apical localization of TLR2 in the intestinal mucosa. Conversely, CNPY3 silencing represses formation of the CNPY3/TLR2 complex and disrupts TLR2 apical distribution, although it fails to change total TLR2 and HuR levels [7,27]. In control cells, TLR2 is primarily distributed at the cell membrane; however, this typical localization is abolished in CNPY3-silenced cells. The levels of TLR2 in membrane fractions prepared from CNPY3-dificent cells decrease significantly when compared with those observed in control cells, while amounts of cytoplasmic TLR2 increase after CNPY3 knockout. Like those observed in CNPY3-deficient cells, silencing of HuR also lowers membrane TLR2 abundance, but it increases the levels of cytoplasmic TLR2. On the other hand, CNPY3 overexpression in HuR-deficient cells rescues cell surface distribution of TLR2, since the membrane TLR2 levels in cells co-transfected with specific HuR siRNA (siHuR) and vector expressing CNPY3 are similar to those observed in cells transfected with control siRNA. Moreover, the disrupted TLR2 distribution alters its functional activity after exposure to TLR2 chemical agonist Pam3csk4 [27]. Treatment of control cells with Pam3csk4 induces TLR2 activation, but this stimulation is prevented by HuR silencing. Overexpression of CNPY3 replenishes the response of HuR-deficient cells to Pam3csk4. These results indicate that decreased CNPY3 by HuR knockout compromises its TLR2 cochaperone function and inhibits the TLR2 apical distribution in the intestinal epithelium. This regulatory role of HuR in subcellular organization of TLR2 through CNPY3-dependent process eventually contributes to the control of secretory autophagy and Paneth cell function.

To define the mechanism by which HuR regulates CNPY3 expression, *Cnpy3* mRNA was found to be a novel target of HuR, and association of HuR with the *Cnpy3* mRNA increases the stability and translation of *Cnpy3* mRNA. Reporters bearing partial transcripts spanning the *Cnpy3* 5′-UTR, CR and 3′-UTR further show that HuR influences CNPY3 expression predominantly via the *Cnpy3* CR but not its 5′-UTR or 3′-UTR, because HuR silencing only represses activity of *Cnpy3*-CR luciferase reporter constructs. HuR commonly interacts with the 3′-UTRs of target transcripts, but it also binds, in some instance, to the CRs of target mRNAs and regulates their fate [17]. Consistently, several HuR-binding motifs such AREs are only identified at the CRs of mouse and human *Cnpy3* mRNA but not at their 5′-UTRs or 3′-UTRs. Additionally, HuR knockout in mice also decreases the levels of other TLR regulatory factors including IRGM and beclin-1, but the exact mechanisms through which HuR regulates expression of IRGM and beclin-1 in IECs and their impact on altered TLR2 localization and subsequent Paneth cell defects in the intestinal epithelium of IE-HuR^−/−^ mice remain to be elucidated.

### 3.4. Regulation of HuR Stability by α4

α4 was first discovered as an immunoglobulin-associated protein in B and T lymphocytes and later identified to be widely distributed in various mammalian cells [61]. α4 is an important regulator of protein phosphatases (PPs), such as PP2A, PP4, and PP6, which account for majority of cellular functions and activity of serine/threonine phosphatases [51,61,62]. Unlike protein kinases, the specificity and activity of PPs are mainly regulated by their complementary proteins. Association of α4 with PP2A displaces the scaffolding (PR65, PP2Aa) and regulatory subunits such as PP2Ab that form the PP2A heterotrimeric complex and thus alters the substrate specificity and enzymatic activity [51]. As a PP2A non-catalytic subunit, α4 governs the specificity and activity of serine/threonine phosphatases and is crucial for controlling cell migration, spreading, proliferation, and apoptosis. Intestinal epithelium-specific deletion of α4 in mice results in defects in Paneth cells, alters crypt proliferation and villus growth, and decreases cell motility along the crypt/villus axis [52]. The intestinal mucosa from α4 knockout mice also exhibits reduced levels of various tight junctions such as claudins and ZO-1 and increased gut permeability.

Interestingly, α4 deficiency decreases the levels of HuR in the intestinal mucosal tissue and cultured IECs by destabilizing HuR protein via a process that involves HuR phosphorylation by IκB kinase α [52]. HuR levels in α4-deficient cells decrease slowly with time after the treatment with cycloheximide, although there are no alterations in HuR levels in control cells exposed to cycloheximide. Additionally, α4 silencing does not alter the stability and cellular level of *HuR* mRNA, suggesting that the reduction in HuR levels in α4-silenced cells results from decreased stability of HuR protein but not from the inhibition of *HuR* gene transcription or altered *HuR* mRNA turnover. Since HuR is subject to ubiquitin-dependent protein degradation that is related to IKKα or PKCα [63,64], further studies show that a reduction in the level of α4 by transfection with its specific siRNA significantly increases phosphorylated IKKα levels without modifying total IKKα abundance. In addition, α4 silencing does not affect the levels of phosphorylated PKCα. Notably, repression of IKKα activity by treatment with its specific chemical inhibitor BAY11-7082 prevents the inhibition of HuR induced by α4 silencing and restores HuR expression level to near normal. Treatment with MG132 also inhibits proteasome and induces HuR levels in α4-deficient cells. Together, these results indicate that α4 improves HuR stability by inhibiting IKKα-mediated HuR phosphorylation in the intestinal epithelium. This process is accomplished via the association of α4 with IKKα that suppresses HuR degradation by decreasing its ubiquitination. Conversely, reducing the levels of α4 destabilizes HuR by intensifying IKKα-mediated HuR phosphorylation and final ubiquitin-dependent proteolysis, thus contributing to the process leading to Paneth cell dysfunction.

### 3.5. Other RBPs in the Regulation of Paneth Cells

CUGBP1 is profoundly expressed in the gut mucosa, and its tissue level, subcellular distribution, and binding affinity for given mRNAs are dramatically affected in various pathophysiological conditions. CUGBP1 binds to many mRNAs via *cis*-elements such as AREs and GREs and this interaction enhances mRNA degradation and/or inhibits translation of target transcripts [53]. Many studies show that CUGBP1 is a repressor of the intestinal epithelium homeostasis [53,54,55]. For example, increased CUGBP1 compromises the gut barrier function by repressing translation of the tight junction occludin [53]. CUGBP1 and HuR competes for binding to the *occludin* mRNA and competitively modulate occludin translation. Increasing the CUGBP1 levels reduces HuR association with *occludin* mRNA and inhibits occludin translation, but elevation of HuR levels abolishes CUGBP1 interaction with *occludin* mRNA and increases occludin translation. Although no available studies directly show the exact role of CUGBP1 in Paneth cells yet, it is possible that CUGBP1 can regulate Paneth cell function and epithelial defense indirectly through association with the HuR in the intestinal epithelium.

AUF1 exhibits a strong affinity for poly (U) and ARE-containing RNAs and is implicated in several aspects of gut mucosal physiology [7,16]. AUF1 regulates the intestinal epithelium homeostasis via transcription factor JunD that is a key modulator of mucosal growth [55,65]. AUF1 binds to *JunD* mRNA, and this association inhibits expression of JunD by increasing degradation of the *JunD* mRNA. Interaction of *JunD* mRNA with AUF1 is negatively modulated by HuR (*JunD* mRNA stabilizer). The levels of cellular polyamines regulate stability of the *JunD* mRNA by changing the competitive binding of AUF1 and HuR to its 3′-UTR [55]. Polyamine depletion increases HuR interaction with *JunD* mRNA but reduces the levels of *JunD* mRNA associated with AUF1, thereby increasing the stability of *JunD* mRNA. HuR silencing promotes AUF1 association with the *JunD* mRNA, decreases the level of HuR/*JunD* mRNA complexes, and causes the *JunD* mRNA to unstable, thus blocking rises in JunD levels in polyamine-depleted cells. Similarly, AUF1 can be involved in the regulation of Paneth cells by inhibiting HuR activity.

**Figure 1:**
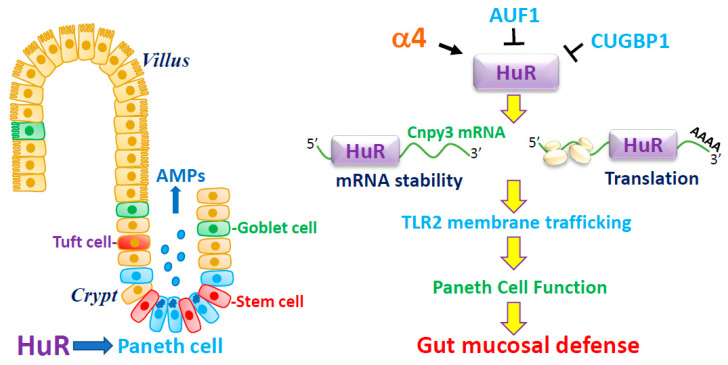
HuR modulates Paneth cell function by controlling membrane distribution of TLR2 through posttrascriptional control of CNPY3. AMPs, antimicrobial proteins. HuR enhances CNPY3 expression by inducing the stability and translation of *Cnpy3* mRNA via association with its 3′-UTR. Induced CNPY3 is essential for the proper membrane localization of TLR2 in the intestinal epithelium, thus sustaining Paneth cell activity. HuR level is positively regulated by α4, but its binding affinity is downregulated by CUGBP1 and AUF1. Disruption of HuR function leads to defective Paneth cells, compromises the intestinal epithelial defense and renewal, and impairs the barrier function.

Taken together, these exciting findings obtained from cultured IECs, intestinal organoids, mice with ablated HuR, and human mucosal tissues indicate a novel model by which HuR plays an important role in the control of Paneth cell function in the small intestinal epithelium (Figure 1). According to this model, HuR improves Paneth cell function by increasing the stability and translation of CNPY3 that is necessary for the proper subcellular distribution of TLR2 on the IEC plasma membrane. In addition, the stability of HuR is positively controlled by α4, but its function can be negatively modulated by CUGBP1 and AUF1. In pathologies such as IBD, disrupted HuR function leads to defective Paneth cells, thus compromising the intestinal epithelial defense and renewal, thus promoting the process of mucosal injury and inflammation.

## 4. Regulation of Paneth Cells by NcRNAs

The mammalian genome is predominantly transcribed to a huge transcriptome of ncRNAs, whereas sequences encoding proteins account only for a small amount of the transcriptional output [66,67]. LncRNAs are defined as transcripts spanning >200 nucleotides in length and involved in various biological processes [67,68]. LncRNAs are widely expressed in different tissues at cell type- and differentiation stages-dependent patterns and they function as molecular signals, scaffolds, or decoys. LncRNAs can also act via cis- and trans-modulatory factors, genomic targeting, and antisense molecules [67]. As a class of small ncRNAs of ~22 nucleotides, miRNAs repress the expression of different genes at the posttranscriptional level by directly interacting with 3′-UTRs of target transcripts [66]. Several high-throughput screening and functional studies have shown that miRNAs are implicated in many cellular processes and play an important role in the pathogenesis of different human diseases. CircRNAs are a novel class of diverse and widespread endogenous ncRNAs that form covalently closed circles [69]. CircRNAs are mainly synthesized from precursor RNAs undergoing splicing, but their 5′ to 3′ termini of splicing byproducts become covalently relegated. Many circRNAs harbor one or several binding locations for a single miRNA, and some harbor binding sites for multiple miRNAs [69,70]. CircRNAs act as RNA sponges or decoys that decrease the amount of freely available miRNAs and they also cooperate with RBPs to jointly modulate gene expression and perform multiple cellular functions [71,72]. Here we focus on the importance of intestinal epithelium enriched ncRNAs, such as lncRNA *H19*, miR-195, and c*ircPABPN1* in the regulation of Paneth cell function (Table 2) and further highlight the mechanisms through which ncRNAs control gene expression through associations with RBPs and/or other ncRNAs.

### 4.1. LncRNA H19 Impairs Paneth Cells and Compromises the Intestinal Barrier Function

*H19* is a 2.3-kb spliced, capped, and polyadenylated ncRNA and transcribed from the conserved imprinted *H19*/*igf2* gene cluster that is localized at human chromosome 11p5.5. *H19* is highly expressed in most organs during early stages of embryogenesis but its expression decreases markedly after birth [73]. Induced levels of *H19* in adult tissues are frequently identified in a wide variety of pathological conditions including inflammation, malignancies, hypoxic environments, and after treatment with estrogen [74,75,76,77]. *H19* functions as a template of primary miR-675 and also acts as an RNA sponge for miRNA let-7. Targeted deletion of *H19* in mice induces growth as noticed by a rise in body weight, which is abolished by transgenic re-expression of the *H19* gene [73]. The function of *H19* in cancer development is intricate, as it can be tumor-suppressor or pro-oncogenic, depending on cellular context of *H19* and tumor types. In the intestinal mucosal tissues, *H19* levels increase remarkably in patients with sepsis and IBD and in murine with acute gut mucosal erosions/injury and inflammation [7,35]. It has been demonstrated that *H19* post-transcriptionally inhibits expression of adherents junction E-cadherin and tight junction ZO-1 in the intestinal epithelium and that this inhibition is mediated through the release of miR-675 embedded in *H19* exon 1 [56]. Induced levels of *H19* leads to the epithelial barrier dysfunction in an in-vitro model using IEC cultures.

The first indication of the importance of *H19* in the regulation of Paneth cells is from our recent observations showing that targeted ablation of the *H19* gene in mice not only increases the function of Paneth and Goblet cells but also stimulates autophagy in the small intestinal epithelium [38]. Although *H19* knockout does not change gut mucosal growth and development in *H19^ΔEx1/+^* (H19^−/−^) mice bearing with a maternal ablation of exon 1 in the *H19* gene, it enhances the functions of Paneth and Goblet cells in the mucosa. The numbers of lysozyme- and mucin 2-positive cells and numbers of lysozyme and mucin 2 granules per cells increase markedly in H19^−/−^ mice compared with control littermates. In an ex-vivo model, ectopically expressed *H19* abolishes an induction in Paneth cells and Goblet cells in the *H19*-deficient intestinal organoids generated from H19^−/−^ mice. These results indicate that *H19* inhibition stimulates function of Paneth and Goblet cells in the small intestinal epithelium.

Moreover, blocking an induction in *H19* levels shields Paneth and Goblet cells from septic stress induced by exposure to cecal ligation and puncture (CLP) in mice [38]. Like observed alterations in wild-type animals, the levels of mucosal tissue *H19* in the small intestine of littermates arise by 24 h after CLP, paired with a remarkable elevation in the levels of miR-675-5p and miR-675-3p. On the other hand, mucosal abundances of *H19* and miR-675 are undetectable in the small intestinal mucosa of H19^−/−^ mice regardless with or without CLP. Although CLP inhibits function of Paneth cells in both control littermate and H19^−/−^ mice, *H19* knockout partially but notably prevents the CLP-induced Paneth cell defects, as shown by more lysozyme-positive cells observed in the intestinal mucosa of H19^−/−^ mice relative to control littermates by 24 h after CLP. In an ex vivo model, exposure to lipopolysaccharide (LPS) also suppresses Paneth cell function as illustrated by a significant reduction in lysozyme-positive cells in the organoids derived from control littermates, but this suppression is mitigated in the organoids generated from H19^−/−^ mice. Likewise, *H19* deletion also protects Goblet cell function in the small intestinal mucosa after exposure to septic stress. Both mucosal tissues from CLP-mice and organoids treated with LPS exhibit decreased numbers of Goblet cells in the presence of *H19*, but the inhibition of Goblet cells by LPS or CLP is prevented by deleting *H19* in both in vivo and ex vivo systems.

Targeted deletion of *H19* also induces autophagy in the small intestinal epithelium [38,78]. Since Paneth cells release lysozyme through secretory autophagy in the intestinal mucosa [26], further studies investigated if *H19* knockout alters autophagy activity after exposure of H19^−/−^ mice to CLP [38]. Basal levels of autophagy proteins ATGs, beclins, and LC3-II in the intestinal mucosa are elevated in *H19*-deficient mice when compared to control littermates. Autophagy activity in the mucosa of littermate mice is inhibited by CLP, but the CLP-induced autophagy inhibition is prevented in H19^−/−^ mice. Despite *H19* deletion not altering gut barrier function without stress, both littermate and H19^−/−^ mice exhibit increased gut permeability by 24 h after CLP. Markedly, however, increased gut permeability induced by CLP in H19^−/−^ mice is significantly less than that observed in littermates. Consistently, the abundances of E-cadherin and ZO-1 proteins also reduce in the intestinal mucosa of littermate mice by 24 h after CLP, but this repression is abolished by the *H19* deletion. In an in vitro system with IEC cultures, *H19* overexpression also inhibits autophagy activation by treatment with a specific pharmacological inducer rapamycin.

**Figure 2:**
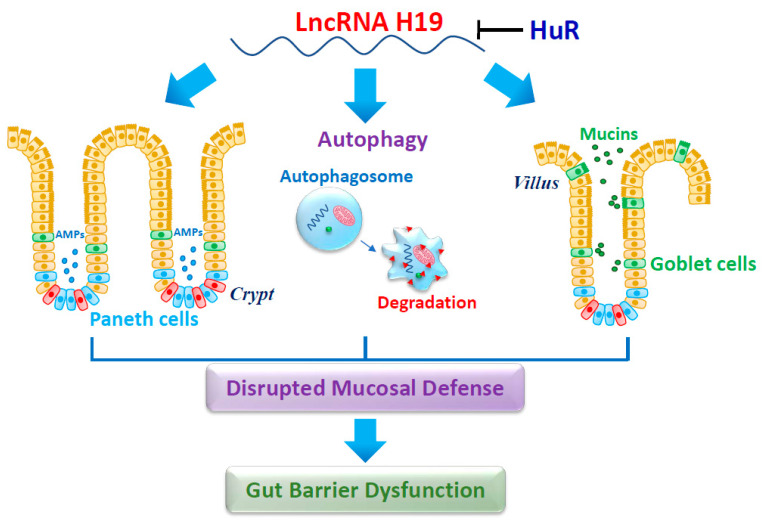
*H19* impairs the intestinal epithelial defense and barrier by repressing function of Paneth and Goblet cells and inactivating autophagy. AMPs, antimicrobial proteins. Targeted deletion of *H19* gene stimulates Paneth and Goblet cell function and promotes autophagy, thus enhancing gut barrier function. HuR directly binds to *H19*, blocks the processing of miR-675 from *H19*, and promotes the epithelial defense and barrier function.

In sum, these interesting results demonstrate that induction in the levels of tissue *H19* by septic stress and/or inflammation impairs the intestinal host defense by disrupting function of Paneth and Goblet cells and suppressing autophagy activity (Figure 2). Conversely, *H19* inhibition bolsters Paneth and Goblet cell function and enhances autophagy, thus sustaining the gut epithelium homeostasis. On the other hand, HuR associates with *H19* and blocks the processing of miR-675 from *H19*. These findings shed a light on how *H19* modulates gut epithelial host defense via Paneth cells in response to pathological stress and exemplify the potential application of targeting therapeutically *H19* and its associating miRNAs and RBPs in patients with gut barrier dysfunction.

### 4.2. MiR-195 Regulates Paneth and Tuft Cells in the Intestinal Epithelium

As an evolutionally conserved miRNA among divergent species, miR-195 is greatly expressed in the intestinal mucosa. miR-195 is involved in several aspects of different biological processes, and it prevents cell proliferation by targeting multiple genes encoding cyclin D1, cyclin-dependent kinase 4 (CDK4), CDK6, SIRT1, WEE1, and ActRIIA [79,80]. Elevating the abundances of miR-195 inhibits IEC migration over the wounded area after injury in an in vitro healing model using cultured IECs [50]. miR-195 suppresses intestinal epithelial repair primarily by destabilizing mRNA encoding STIM1, a protein pivotal for stored-operated Ca^2+^ influx in IECs after injury. Induced miR-195 also represses insulin-like growth factor (IGF) signaling by inhibiting translation of the IGF2-receptor [81]. On the other hand, the levels of cellular miR-195 in the intestinal epithelium are negatively regulated by lncRNA *uc.173* through stimulation of primary miR-195 degradation [57], and its binding affinity for given mRNAs is abolished by HuR [50].

A recent study utilizing a transgenic gain-of-function approach demonstrates that miR-195 plays a critical role in the regulation of Paneth and Tuft cell function [46]. Transgenic expression of miR-195 (miR195-Tg) in the intestinal epithelium results in dysfunction of both Paneth and Tuft cells in mice. Although double cortin-like kinase 1 (DCLK1)-positive cells (Tuft cells) are primarily interspersed at the villous regions, but Paneth cells are only distributed at the base of the crypt areas in the intestine. However, the numbers of Paneth and Tuft cells in the intestinal mucosa decrease significantly in miR195-Tg mice compared to control littermates. Furthermore, the abundances of lysozyme and DCLK1 proteins in the intestinal mucosa are also reduced in miR195-Tg mice as determined by immunoblotting analysis. Similar to the in vivo findings, there are evident irregularities in Paneth and Tuft cells in the intestinal organoids derived from the miR195-Tg mice. In the organoids isolated from littermates, Paneth and Tuft cells are enriched but they decrease significantly in the organoids generated from miR195-Tg mice. In contrast, intestinal epithelium-specific miR-195 overexpression fails to influence Goblet cell function. The structure and numbers of Goblet cells in the mucosa of miR195-Tg mice are indistinguishable from those in control littermates. Moreover, transgenic miR-195 overexpression does not alter enterocyte differentiation in the epithelium as examined by villin immunostaining assay.

Unexpectedly, locally elevating the levels of miR-195 in the intestinal epithelium does not affect mucosal growth in miR-195-Tg mice [46,57]. There are no substantial disparities in the histological characteristics of the intestinal mucosa, population of proliferating (BrdU-positive) cells in the crypts, proliferation marker proteins such as PCNA and Ki67, and the lengths of crypts and villi between littermate mice and miR195-Tg mice. Growth rate of the intestinal organoids isolated from miR195-Tg mice exhibit no significant change compared with that from littermate mice. Increased levels of miR-195 does not directly alter gut permeability in miR195-Tg mice but increase susceptibility of the gut barrier to LPS-induced stress as examined by FITC-conjugated dextran assays. Increased gut permeability is higher in miR195-Tg mice than that observed in littermate mice after exposure to the same dose of LPS. Additionally, elevation of the local miR-195 level in the intestinal mucosal tissues does not cause apoptosis. These results reveal that increasing the levels of tissue miR-195 specifically impairs function of Paneth and Tuft cells in the small intestinal epithelium without effect on enterocytes and Goblet cells.

The mechanism underlying defective Paneth cells in miR195-Tg mice remains largely unknown, but it may be related to miR-195 interaction with HuR. As pointed out above [27,52], HuR is necessary for maintaining Paneth cell function, while miR-195 has antagonizing impact on HuR-induced stability and translation of target mRNAs [46]. For example, miR-195 overexpression induces destabilization of the *Stim1* mRNA by increasing the levels of *Stim1* transcripts in P-bodies [50], where untranslated mRNAs are sorted for degradation [15]. In contrast, elevating the levels of HuR abolishes miR-195-induced destabilization of the *Stim1* mRNA by reducing the *Stim1* mRNA recruitment to P-bodies. In support to this notion, miR-195 down-regulates Tuft cells by repressing DCLK1 translation through direct association with the *Dclk1* mRNA, while HuR competes with miR-195 for interaction with *Dclk1* mRNA and enhances DCLK1 expression in cultured IECs [46].

### 4.3. CircRNAs Are Novel Regulators of the Intestinal Epithelium Homeostasis

*CircPABPN1* is transcribed from the *PABPN1* gene and implicated in the modulation of Paneth cell function by regulating autophagy in the small intestinal mucosa through interaction with HuR [32]. *CircPABPN1* is a notable HuR target circRNA and increasing the levels of *circPABPN1* blocks HuR association with the *PABPN1* mRNA in HeLa cells [58]. Consistently, *circPABPN1* also directly interacts with HuR in cultured IECs. Ectopically expressed *circPABPN1* suppresses HuR association with the *Atg16l1* mRNA and represses the expression of ATG16L1 without effect on cellular levels of HuR and ATG5. Furthermore, overexpression of HuR rescues expression of ATG16L1 in IECs transfected with *circPABPN1* expression vector, while *circPABPN1* induction and HuR inhibition synergistically repress the expression of ATG16L1. Since there are no potential binding sites for *circPABPN1* in the *Atg16l1* mRNA, *circPABPN1* represses ATG16L1 translation by inhibiting HuR binding to the *Atg16l1* transcript. Importantly, human intestinal tissues from patients with inflammation/injury and growth inhibition exhibit elevated abundances of *circPABPN1* and lowered HuR levels, along with a decrease in both ATG16L1 and Paneth cells [32]. Based on fact that HuR is crucial for normal function of Paneth cells and that HuR targets multiple transcripts, it is likely that altered interaction between *circPABPN1* and HuR plays a critical role in controlling Paneth cell function in response to pathophysiological stress.

Recently, we have reported that *circHIPK3* promotes intestinal epithelial homeostasis by reducing miR-29b function [36]. *circHIPK3* expression levels in the intestinal mucosal tissues are significantly altered in CLP-mice relative to sham mice, and human intestinal mucosa obtained from patients with sepsis and IBD display reduced *circHIPK3*, along with defective Paneth cells, injury/erosions, massive inflammation, and mucosal atrophy [27,38]. Increasing the levels of *circHIPK3* enhances intestinal epithelial repair after wounding, whereas c*ircHIPK3* silencing represses epithelial recovery. *circHIPK3* silencing also inhibits growth of IECs and intestinal organoids, but *circHIPK3* overexpression promotes intestinal mucosal growth in mice. Mechanistically, c*ircHIPK3* interacts with miR-29b and inhibits miR-29 activity, thus increasing expression of Rac1, Cdc42, and cyclin B1 in IECs after wounding [36,59]. Although the exact role of *circHIPK3* in regulating Paneth cell function remains unknown, these findings indicate that *circHIPK3* is necessary for sustaining homeostasis of the intestinal epithelium by enhancing intestinal epithelial repair and promoting mucosal renewal via interaction with miR-29b.

## 5. Conclusions

Paneth cells play an important role in mucosal physiology by their specific location and large repertoire of effector molecules in the small intestine. The contributions of Paneth cells to gut mucosal development, host defense, and rapid renewal are crucial for the epithelium homeostasis. Important secretory products by Paneth cells are antimicrobial peptides/proteins that are necessary for functional interactions between the epithelium and the microbiome. Paneth cells are also intimately implicated in creating the ISC niche located at the crypt base of small intestine, which is critical for constant mucosal renewal and morphogenesis of the crypt-villus axis. Although Paneth cell function is tightly regulated by multiple factors at different levels, Paneth cell defects are common in gut mucosal pathologies and are correlated with many intestinal disorders such as IBD, bacterial infection, cancers, and necrotizing enterocolitis.

Control of mRNA turnover and translation by RBPs and ncRNAs represents a critical layer of intricacy regulating intestinal epithelial host defense and homeostasis. RBPs, lncRNAs, and miRNAs are shown to act as multifunctional molecules in maintaining the intestinal epithelial integrity, although studies defining the roles of circRNAs in the gut mucosal physiology are still limited. The findings summarized here provide strong evidence that RBPs and ncRNAs participate in the regulation of Paneth cells. HuR is required for Paneth cell function by maintaining apical localization of TLR2 through post-transcriptional regulation of CNPY3. *H19*, miR-195, and *circPABPN1* also regulate function of Paneth cells directly or indirectly, but the exact mechanisms underlying these ncRNAs remain to be fully investigated. Interestingly, HuR can interact with many ncRNAs to jointly modulate their binding affinity and biological functions. Paneth cell differentiation and function are dependent on a dynamic balance between various RBPs and ncRNAs, whereas deregulation of given RBPs and ncRNAs leads to Paneth cell defects, thus contributing to the pathogenesis of various gut mucosal diseases in humans. These exciting findings assist us in understanding how Paneth cells preserve their function under pathologic conditions and how altered Paneth cells by RBPs and ncRNAs act as a potential therapeutic target to protect the integrity of intestinal epithelium in patients with critical illnesses.

## Figures and Tables

**Table 1:** Role of Paneth cells in the intestinal epithelium homeostasis and diseases.

Function	Mechanisms	References
**Host defense**	AMPs: Lysozyme α-defensin C-type lectins Phospholipase A2 Reg-III MMP-7 CRIP Xanthine oxidase Autophagy Apoptosis	Bel et al. [26]; Xiao et al. [27] Ayabe et at. [25]; Salzman et al. [28]; Porter et al. [29] Riba et al. [5] Boya et al. [8] Bel et al. [26] Peterson et al. [30] Yang et al. [21] Lueschow et al. [13] Clevers et al. [11] Bel et al. [26]; Dikic et al. [31]; Li et al. [32] Gunther et al. [33]; Giammanco et al. [34]
**Mucosal renewal**	Stem cell niches Niche signaling (Wnt, Notch)	Beumer et al. [6]; Clevers et al. [11] Sato et al. [12]
**Impact on diseases**	IBD Bacterial Infection Sepsis Cancers NEC	Yang et al. [21]; Geng et al. [35] Xiao et al. [36] Riba et al. [5]; Beumer et al. [37]; Salzman et al. [28] Yu et al. [38] Chatterji et al. [39]; Giammanco et al. [34] Torow et al. [2]; Gunther et al. [33]

**Table 2:** Roles of RBPs and ncRNAs in the regulation of Paneth cell function.

Names	Functions	Targets	References
**RBPs** **HuR**	↑ Paneth cell function	Enhancing TLR2 membrane distribution and activity via CNPY3 Inhibiting miR-195 activity Interacting with α4 via IKKα	Xiao et al. [7,27] Delgado et al. [49] Zhuang et al. [50] Kong et al. [51] Chung et al. [52]
**CUGBP1**	↓ Paneth cell function	Competing with HuR to interact with target mRNAs	Yu et al. [53] Yu et al. [54]
**AUF1**	↓ Paneth cell function	Inhibiting HuR binding to target mRNAs	Zou et al. [55]
**NcRNAs** ***H19***	↓ Paneth cell function	Inhibiting autophagy, reducing lysozyme, and impairing barrier	Yu et al. [38] Zou et al. [56]
***uc.173***	↑ Paneth cell function	Increasing miR-195 degradation	Xiao et al. [57]
***miR-195***	↓ Paneth and Tuft cells	Inhibiting HuR binding to mRNAs	Kwon et al. [46]
***circPABPN1***	↓ Paneth cell function	Preventing HuR binding to mRNAs Inhibiting ATG16L1 translation	Abdelmohsen et al. [58] Li et al. [32]
***circHIPK3***	↑ Paneth cell function	Enhancing epithelial homeostasis via interacting with miR-29b	Xiao et al. [36] Ni et al. [59]

## Data Availability

Not applicable.

## Abbreviations

IECs: intestinal epithelial cells; ncRNAs, noncoding RNAs; lncRNAs, long ncRNAs; RBPs, RNA-binding proteins; IBD, inflammatory bowel diseases; ATGs, autophagy-related genes; TLR2, Toll-like receptor 2; RRM, RNA recognition motif; LC3, light chain 3; miRNA, microRNA; CLP, cecal ligation and puncture; CNPY3, canopy3; CRs, coding regions; UTRs, untranslated regions; DSS, dextran sulfate sodium; circRNAs, circular RNAs; HuR, human antigen R (ELAVL1); HuB, human antigen B (ELAVL2); HuC, human antigen C (ELAVL3); HuD, human antigen D (ELAVL4).
